# Bacitracin-Ag Nanoclusters as a Novel Antibacterial Agent Combats *Shigella flexneri* by Disrupting Cell Membrane and Inhibiting Biofilm Formation

**DOI:** 10.3390/nano11112928

**Published:** 2021-11-01

**Authors:** Lin Wang, Liu Liu, Xiaotong Zhou

**Affiliations:** College of Food Engineering and Nutritional Science, Shaanxi Normal University, Xi’an 710119, Shaanxi, China; Wangliner417@163.com (L.W.); Auty_zhou@126.com (X.Z.)

**Keywords:** Ag, nano, bacitracin, *Shigella flexneri*, inhibition, biofilm

## Abstract

A novel nanomaterial Bacitracin-Ag Nanoclusters (Bacitracin-AgNCs) was formed to achieve a better antibacterial effect on *Shigella flexneri* which poses a serious threat to human health. In the current study, X-ray photoelectron spectrometer (XPS), Fourier transform infrared (FTIR), field-emission scanning electron microscopy (FESEM), high resolution transmission electron microscopy (HR-TEM) and thermal gravimetric analysis (TGA) were used to characterize the properties of composited Bacitracin-AgNCs. Furthermore, the inhibitory effects of Bacitracin-AgNCs against *S. flexneri* were explored, and the inhibition mechanism was discussed in terms of its aspects of cell membrane ravage, ATPase activity decline and biofilm inhibition. The results reveal that the minimum inhibitory concentration (MIC) and minimum bactericidal concentration (MBC) of Bacitracin-AgNCs against *S. flexneri* were 0.03 mg/mL and 4 mg/mL. Bacitracin-AgNCs may cause irreversible impairment to cells and greatly change the cell morphology. The cell membrane integrity of *S. flexneri* was destroyed with changes in the characteristics of membrane permeability and intracellular substances leakage. Moreover, our study further proved that Bacitracin-AgNCs significantly inhibited the formation of *S. flexneri* biofilms and reduced the number of viable bacteria in biofilm. These findings provide a potential method for the exploitation of organic composite nanomaterials as a novel antimicrobial agent and its application in the food industry.

## 1. Introduction

*Shigella* spp. are facultatively anaerobic, nonsporing and atrichous gram-negative short rod-shaped pathogenic bacteria, which can cause different degrees of dysentery [1]. The genus consists of four species: *Shigella flexneri*, *Shigella sonnei*, *Shigella boydii* and *Shigella dysenteriae*. *Shigella flexneri* is one of the uppermost pathogens causing foodborne bacterial infections. Related studies have found that *S. flexneri* has been detected in the food industry, including in meat products, dairy products and some markets and workshops [2,3]. *S. flexneri* has a stable biofilm structure, which is a bacterial aggregation of extracellular polymeric substances (EPS) composed of polysaccharides, proteins, DNA and lipids [4]. The biofilm formation is a key virulence factor for microorganisms causing chronic infections. At present, the main treatment of *Shigella* is antibiotic therapy. However, the therapeutics of bacterial infections becomes more difficult with the emergence of resistant strains and biofilms. Therefore, it is of great significance to develop new and effective methods to destroy the biofilm integrity and prevent the growth of *S. flexneri*.

Currently, the treatment of bacterial infections based on nanomaterial antimicrobial agents has become a research hotspot. Recently, metal nanoclusters have attracted increasing attention in the field of biomedical science due to their unique physical and chemical properties, such as high surface-to-volume ratio, strong fluorescence and discrete energy levels [5]. At the same time, the discovered antibacterial properties of nanomaterials have also turned them into prospective candidates to help enhance the efficiency of traditional small molecule antibiotics [6].

Generally, metal nanoparticles can be synthesized by various chemical and physical methods. However, the materials used in these chemical methods, such as organic reagents and stabilizers, as well as radiometry in physical methods, have obvious flaws in particle aggregations and environmental harm [7]. It is an imperative demand to explore simple and environmentally friendly nanomaterials with enhanced activities. Based on this, the biological template method has become a feasible new research method, one which has the advantages of good dispersity, simple synthesis and environmentally protective ingredients [8]. Many bioactive molecules, such as proteins [9], DNA [10], viruses [11] and so on, have been successfully applied to the preparation of nanomaterials.

Bacitracin, with the characteristics of water solubility, low-toxicity, and heat stability, is a polypeptide antibiotic extracted from *Bacillus subtilis* and *Bacillus licheniformis*. The bacitracin can self-assemble into larger molecules, thus better binding with metal ions and further forming unique morphology in the process of co-cultivation with metal salt solution [12]. As a kind of well-known antibacterial agent, silver can exert antimicrobial action through various kill mechanisms, like membrane damage and DNA damage, also, it may coordinate with different antibacterial agents to form composite silver nanomaterials so as to stimulate better antibacterial effects [13]. Silver nanocomposites have highly adjustable antibacterial efficiency with strong antifungal activity against some Gram-positive and Gram-negative bacteria [14]. Additionally, compared with most narrow-spectrum antibiotics, the highly antibacterial effect shown in silver nanoparticles is significantly more apparent on multidrug-resistant (MDR) strains. Based on this, the development of a new type of nanomaterial with biological molecules as templates has become a trend.

Nevertheless, little report has been made on the synthesis of composite silver nanomaterials with the combination of the bacitracin template and silver ions, and the antibacterial effects as well as the mechanisms of synthetic nanomaterials against *S. flexneri* are poorly researched. Hereby, we chose bacitracin as an embedding agent to develop a water-soluble nanomaterial via encapsulating silver ions and characterized the properties of the material. The composite nanomaterial showed strong antimicrobial action on *S. flexneri*. Moreover, we depicted the inhibition effects of Bacitracin-AgNCs on the biofilm formation of *S. flexneri*, clarifying the inhibitory mechanisms of the nanomaterial on *S. flexneri*.

## 2. Materials and Methods

### 2.1. Chemical Reagents and Materials

Bacitracin was obtained from BidePharm Technology Co., Ltd. (Shanghai, China), and levofloxacin (LFX) was purchased from JingBo Biotechnology Co., Ltd. (Xi’an, China). All other chemicals were of analytical grade. *S. flexneri* ATCC 12022 was obtained from the Food Safety and Hygiene Laboratory (Shaanxi Normal University, Xi’an, China). Before each experiment, the *S. flexneri* strains were shake-cultured in Luria–Bertani (LB) broth for 18 h at 37 °C.

### 2.2. Synthesis, Purification and Characterization of Bacitracin-AgNCs

The synthesis of Bacitracin-AgNCs was performed according to a method described previously [15] with certain modification. Briefly, 1 mL bacitracin (10 mg/mL) was added to 1.6 mL AgNO_3_ (17 mM) to synthesize Bacitracin-AgNCs. Then, 200 µL NaOH (1 M) and 20 µL NaBH_4_ (2 mM) were introduced to adjust the alkaline conditions (pH = 12) and enhance stability, respectively, and the mixture was incubated at 37 °C for 3.5 h. The sample was collected by centrifugation (10,000 rpm, 15 min) and the supernatant was filtered by the membrane of 0.22 μm. Then, the nanoclusters were obtained by dialysis (cut-off of 300 MWCO) in deionized water for 24 h. Finally, the nanoclusters powder was acquired by lyophilization and stored in a vacuum dryer for subsequent use.

For the characterization of Bacitracin-AgNCs, we firstly used UV300 UV-VIS spectrophotometer (Yimai Instrument Technology Co., Ltd., Shanghai, China) to record the absorbance of the nanoclusters, the solution was properly diluted with distilled water before measurement. A fluorescence spectrophotometer (RF-6000, Shimadzu, Kyoto, Japan) was used to identify the fluorescence properties. Elemental and functional groups of Bacitracin-AgNCs were analyzed by ESCALAB Xi+ X-ray photoelectron spectrometer (XPS) (Thermo Fisher Scientific, Waltham, MA, USA) and Fourier transform infrared spectrometer (FTIR) (Bruker, Karlsruhe, Germany). The surface structures of Bacitracin-AgNCs and bacitracin were observed using SU-8020 field-emission scanning electron microscopy (FESEM) (Hitachi, Tokyo, Japan), the microcrystalline powders of two samples were fixed on the conductive adhesive of the sample table and sprayed with gold for 10 s for observation. High resolution transmission electron microscopy (HR-TEM) (JEOL, Tokyo, Japan) and a dynamic light scattering (DLS) instrument (BI-90Plus, Brookhaven, NY, USA) were used to observe the morphology and measure the particle size of the nanoclusters. Thermal gravimetric analysis (TGA) was performed by Q600 Thermo analyzer Systems (TA Instruments, waters, MA, USA) to determine thermal stability of Bacitracin-AgNCs in the condition of heating the samples from 25 to 600 °C at a heating rate of 10 °C/min in a nitrogen atmosphere.

### 2.3. Antibacterial Activity on S. flexneri

#### 2.3.1. LB Liquid Medium Turbidity Assay

In order to visually observe the antibacterial activity of Bacitracin-AgNCs against *S. flexneri*, we performed the turbidity experiment in LB liquid medium according to a previous research method [16]. *S. flexneri* in a logarithmic growth phase was respectively added to LB broth containing Bacitracin-AgNCs, bacitracin and LFX (the final concentration was 10 mg/mL), and the mixed solution was shake-cultured at 37 °C for 16 h. The LB broth without drugs treatment served as blank control. Finally, the optical density of the corresponding bacterial suspension at 600 nm (OD_600_) was measured at 0 and 16 h.

#### 2.3.2. Determination of Minimum Inhibitory Concentration (MIC) and Minimum Bactericidal Concentration (MBC)

Stock solution of Bacitracin-AgNCs at the concentrations of 16 mg/mL was diluted into 10 tubes containing sterile LB Broth to make the concentrations from 0.016 to 8 mg/mL. Then, *S. flexneri* in a logarithmic growth phase (approximately 10^7^ CFU/mL) was inoculated into every tube and cultured at 37 °C for 24 h under shaking conditions (160 rpm). MIC value was the lowest concentration of Bacitracin-AgNCs solution without t visible bacterial growth in LB broth. The cells prepared in the test tubes were dispersed on Salmonella Shigella agar (SS) plates, and the MBC value was regarded as the lowest concentration of Bacitracin-AgNCs solution, where there were no visible bacterial colonies on the surface of SS agar plates.

#### 2.3.3. Growth Curve Assay

The powder of Bacitracin-AgNCs was dissolved in sterile LB broths to acquire the final concentrations of 1/16 MIC, 1/8 MIC, 1/4 MIC, 1/2 MIC, and MIC. LB without Bacitracin-AgNCs was used as a control. LFX and bacitracin solution prepared with the same concentration of MIC were used as positive controls. Then, *S. flexneri* in logarithmic growth phase (approximately 10^7^ CFU/mL) was inoculated into each LB broth and the mixture was incubated at 37 °C and 160 rpm for 24 h. The growth of bacteria was recorded by a Thermo Scientific Multiskan Spectrum (Thermo Fisher Scientific, Waltham, MA, USA) at 600 nm at 2 h intervals.

### 2.4. Antibacterial Mechanism of Bacitracin-AgNCs against S. flexneri

#### 2.4.1. Release of Cell Constituents

Cell integrity was examined by determining the leakage of cell constituents according to previously published methods [17,18], with minor modifications. Bacitracin-AgNCs (at final concentration of 0 (control), 1/4 MIC, 1/2 MIC, MIC, MBC) was added into *S. flexneri* in a logarithmic growth phase (approximately 10^7^ CFU/mL). The mixtures were incubated at 37 °C for 8 h under 180 rpm. 1 mL suspension was absorbed from the mixtures every hour and centrifuged at 8000 rpm for 5 min, and the absorbance of supernatant was measured at 260 nm by a UV-VIS spectrophotometer (Yimai Instrument Technology Co., Ltd., Shanghai, China).

#### 2.4.2. Membrane Permeability Assays

The cell membrane permeability of *S. flexneri* cells was determined by measuring the release of *β*-galactosidase activity into the culture medium with ONPG as a substrate [19]. *S. flexneri* grown to logarithmic phase in LB medium containing 2% lactose was collected by centrifugation at 8000 rpm for 10 min, washed three times and resuspended (to give 10^7^ CFU/mL) in sterile PBS. Then, Bacitracin-AgNCs (at final concentration of 0 (control), 1/4 MIC, 1/2 MIC, MIC, MBC) was added into the *S. flexneri* suspensions. After 8 h of incubation at 37 °C, the solution was centrifuged at 8000 rpm for 5 min, and *o*-nitrophenyl-*β*-D-galactopyranoside (ONPG, at a final concentration of 1.5 mM) was added to the supernatant. The mixture was incubated at 37 °C for 30 min. The cell membrane permeability was determined at 420 nm by a UV-VIS spectrophotometer (Yimai Instrument Technology Co., Ltd., Shanghai, China) every hour.

#### 2.4.3. Confocal Laser Scanning Microscopy (CLSM) Assay

To detect bacterial viability and membrane integrity, the living/dead staining of bacterial cells was performed as described in a previous study with slight modifications [20]. *S. flexneri* suspension in logarithmic growth phase (about 10^7^ CFU/mL) was centrifuged, the supernatant was removed and the obtained cells were resuspended with sterile PBS. Then, the *S. flexneri* suspension was cultured with Bacitracin-AgNCs at MIC and MBC for 6 h at 37 °C, and the inoculum without antimicrobial agents as a control. After treatment, the bacteria cells were washed twice with sterile PBS and suspended again. Subsequently, 100 μL of cell suspension was dropped onto a slide glass, also, 5 μL of acridine orange (AO) and 5 μL of ethidium bromide (EB) were added into the cell suspension. After blowing gently and blending the solution, the slide was covered and the cells were stained at room temperature for 2 min. Samples were observed by a confocal laser scanning microscope (CLSM) (Olympus Corporation, Tokyo, Japan).

#### 2.4.4. Determination of Cellular Na^+^ K^+^- and Ca^2+^ Mg^2+^-ATPase Activity

The *S. flexneri* cells in logarithmic growth phase (approximately 10^7^ CFU/mL) were collected and treated with Bacitracin-AgNCs (at final concentrations of 0 (control), MIC, MBC) at 37 °C for 0.5–2 h. Subsequently, the effects of Bacitracin-AgNCs on Na^+^ K^+^- and Ca^2+^ Mg^2+^-ATPase activity of *S. flexneri* were determined by an ATPase assay kit (Sangon Biotech, Shanghai, China).

#### 2.4.5. Field-Emission Scanning Electron Microscopy (FESEM) Analyses

*S. flexneri* cells were treated with Bacitracin-AgNCs (0 (control), MIC, MBC) and incubated at 37 °C for 6 h. Part of the culture medium was poured out and centrifuged (5000 rpm) at 4 °C for 10 min to obtain cell aggregates. The harvested cells were rinsed three times with sterile PBS and fixed in 2.5% glutaraldehyde for 6 h at 4 °C to maintain cell aggregation. Then, the samples were dehydrated in gradient ethanol (25, 50, 75, 95, 100%) for 10 min. The dehydrated samples were sprayed with gold and tested under an FESEM (SU8220, Hitachi, Tokyo, Japan).

### 2.5. Inhibition of Biofilm Formation by Bacitracin-AgNCs

#### 2.5.1. Biofilm Formation and Quantitative Crystal Violet Assay

The formation of biofilm was quantitatively determined in 96-well polystyrene plates (JET BIOFIL, Guangzhou, China) with crystal violet staining method introduced by [21], with some modifications. The powder of Bacitracin-AgNCs was dissolved in sterile 1/8 LB medium to make final concentration of 1/16 MIC, 1/8 MIC, 1/4 MIC, 1/2 MIC and MIC. LFX and bacitracin solutions prepared with the same concentration of MIC were used as positive controls, and the sterile 1/8 LB broth without antibacterial agents served as a blank control. Then, 200 μL of the above antibacterial agents and *S. flexneri* (approximately 10^7^ CFU/mL) were added to a 96-well polystyrene microtiter plate and incubated at 37 °C for 24 h under static conditions. After biofilm formation, the suspension was gently removed and the 100 μL of sterile PBS (0.01 M) was used to remove planktonic bacteria. The samples were fixed with methanol for 15 min and then stained with 200 μL of 1% crystal violet solution at 37 °C for 15 min in a dark environment. Finally, the stained biofilms were dissolved in 200 μL of 95% ethanol. The OD values at 570 nm were obtained by a Thermo Scientific Multiskan Spectrum. The following formula was used to calculate the percentage of biofilm formation inhibition:

(1) $$\mathrm{The}\mathrm{percentage}\mathrm{inhibition}(\%)=\frac{OD_{control}-OD_{sample}}{OD_{control}}\times 100$$

#### 2.5.2. Environment Scanning Electron Microscopy (ESEM) Analysis

*S. flexneri* suspension (approximately 10^7^ CFU/mL) was incubated in 1/8 LB broth with Bacitracin-AgNCs (0 (control), 1/16 MIC, 1/8 MIC, 1/4 MIC, 1/2 MIC, MIC), LFX and bacitracin to form biofilms on glass coverslips (4 mm × 4 mm) in a 24-well polystyrene microtiter plate for 48 h at 37 °C. After biofilm formation, the samples were fixed in fixative (2.5% glutaraldehyde) for 6 h at 4 °C, then washed with PBS and dehydrated with a series of ethanol concentrations (25, 50, 75, 95, 100%) for 10 min. Finally, the dehydrated specimens were coated with gold and observed by an ESEM (FEI-Quanta 200, Hillsboro, OR, USA) [22].

### 2.6. Statistical Analysis

All experiments were performed in triplicate, and the data were indicated as arithmetic mean ± standard deviation. Statistical analysis was performed using SPSS software (version 22.0, SPSS, Chicago, IL, USA). One-way analysis of variance (ANOVA) was used to calculate the significant differences. *p* < 0.05 was considered statistically significant.

## 3. Results

### 3.1. Synthesis and Fabrication of Bacitracin-AgNCs

**Figure 1:**
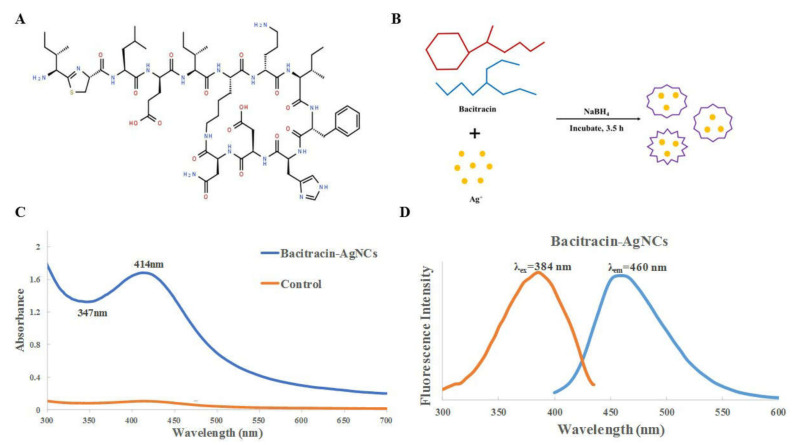
(**A**) Chemical structure of bacitracin. (**B**) Scheme for producing Bacitracin-AgNCs. (**C**) UV-VIS absorption spectra of Bacitracin-AgNCs. (**D**) Fluorescence spectra (excitation, emission) of Bacitracin-AgNCs.

Bacitracin is a cyclic peptide, consisting of 12 amino acid residues (Figure 1A), which has good biocompatibility. The synthesis mechanism of Bacitracin-AgNCs is shown in Figure 1B. The formation of silver clusters can be achieved by reduction of silver ions with NaBH_4_. In this process, bacitracin as an entrapment agent can cover different surfaces and connect inorganic molecules with biological molecules. Therefore, bacitracin embeds silver atoms and couples to form a new nano antibacterial agent under synthetic conditions.

### 3.2. Characterization of Bacitracin-AgNCs

#### 3.2.1. UV-VIS Spectra and Fluorescence Spectroscopy

The UV-VIS spectra and fluorescence spectroscopy of Bacitracin-AgNCs are shown in Figure 1. Bacitracin-AgNCs has an obvious absorption peak at 414 nm. According to a previous report [23], the peak near 420 nm in the spectra is a typical peak of AgNPs, indicating the successful preparation of nanoclusters. The characteristic absorption peaks of Bacitracin-AgNCs correspond to the reported spherical nanoparticles AgNPs-2 with similar spherical shapes [24]. The optimum excitation and emission wavelengths of Bacitracin-AgNCs are 384 nm and 460 nm, respectively.

#### 3.2.2. Fourier Transform Infrared Spectrometer (FT-IR) and X-ray Photoelectron Spectroscopy (XPS)

**Figure 2:**
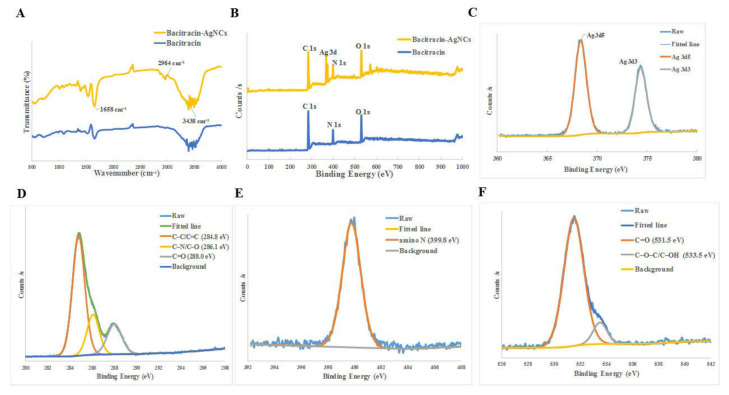
(**A**) FTIR spectrums of bacitracin and Bacitracin-AgNCs. (**B**) XPS full scan survey of bacitracin and Bacitracin-AgNCs. (**C**) Binding energy spectra of Ag 3d. (**D**–**F**) High-resolution spectra of C 1s, N 1s and O 1s.

We use FTIR and XPS to analyze the functional groups and components of the Bacitracin-AgNCs in detail. Firstly, the surface groups of synthesized Bacitracin-AgNCs were examined by FTIR (Figure 2A). Briefly, the peak at 3438 cm^−1^ was the result of characteristic absorption of the O–H stretching vibration, while the peak at 1658 cm^−1^ was C=O stretching vibration, indicating that carboxylic groups existed in synthesized nanoclusters. Meanwhile, the characteristic absorption of 2964 cm^−1^ revealed the N–H stretching vibration [25,26]. X-ray photoelectron spectroscopy (XPS) survey were used to further elucidate the element components of Bacitracin-AgNCs. The full spectra shown in Figure 2B revealed that there were 3 typical peaks of C 1s (285 eV), N 1s (400 eV) and O 1s (531 eV) in bacitracin as well as 4 obvious peaks of C 1s (285 eV) (67.74%), N 1s (400 eV) (11.70%), O 1s (532 eV) (17.65%) and Ag 3d (368.4, 374.4 eV) (2.91%) in Bacitracin-AgNCs, indicating the successful combination of silver atom and bacitracin in nanoclusters. In the high-resolution spectra, the C 1s band could be deconvoluted into three peaks (Figure 2D), including sp^2^ carbons (C=C, 284.8 eV), sp^3^ carbons (C–O/C–N, 286.1 eV) and carbonyl carbons (C=O, 288.0 eV), respectively [27]. The N 1s band contained a peak representing amino N at 399.8 eV (Figure 2E). Also, the O 1s band of O 1s spectrum could be divided into two peaks (Figure 2F) at 531.5 eV and 533.5 eV, which pertained to C=O, C–O–C and C–OH groups, respectively [28]. Overall, Bacitracin-AgNCs contained the characteristic functional groups of –OH, –COOH, C=O and –NH_2_. The above FTIR and XPS results suggested that Bacitracin-AgNCs involved *π*-conjugated and oxidation domains, as well as amorphous regions at the same time [29]. In addition, the binding energies of Ag 3d (368.4, 374.4 eV) of nanoclusters were approaching that of silver (368.0, 374.0 eV) (Figure 2C), proving the complete reductions of silver ions [30].

#### 3.2.3. FESEM and HR-TEM of Bacitracin-AgNCs

**Figure 3:**
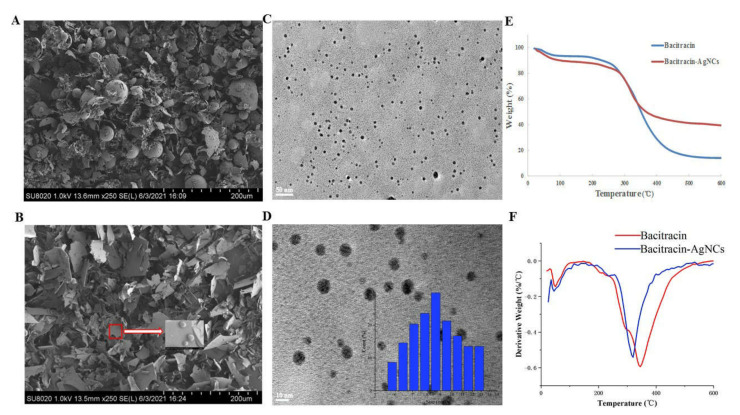
(**A**) FESEM images of bacitracin and (**B**) Bacitracin-AgNCs. (**C**,**D**) HR-TEM images of Bacitracin-AgNCs (inset: histogram particle size distribution of Bacitracin-AgNCs by DLS). (**E**) Thermal gravimetric analysis (TGA) curves of bacitracin and Bacitracin-AgNCs. (**F**) Differential thermogravimetry (DTG) curves of bacitracin and Bacitracin-AgNCs.

The surface structures of nanoclusters were observed by field-emission scanning electron microscopy (FESEM). It could be seen that bacitracin had a relatively dense structure (Figure 3A), while the surface of Bacitracin-AgNCs was loose (Figure 3B). To further characterize the proposed nanoclusters, we used high resolution transmission electron microscopy (HR-TEM) to observe the morphology of Bacitracin-AgNCs. The result shown in Figure 3C,D suggest that Bacitracin-AgNCs was spherical and had good dispersion in solution with an average size of 10 nm, which was proved by DLS result.

#### 3.2.4. Thermogravimetric Analysis (TGA)

Examination of the thermal stability of Bacitracin-AgNCs was carried out by a TGA instrument and the result is shown in Figure 3E. For the bacitracin and Bacitracin-AgNCs, the weight loss observed from the curves can be divided into two stages. Firstly, the slight decline occurring below 100 °C was caused by evaporation of the water retained in the material. Then, the second weight loss stage (temperature ranging from 200 to 400 °C) could be induced by the thermal decomposition of raw materials. Only one decomposition peak for both bacitracin and Bacitracin-AgNCs (Figure 3F) described the compatibility between bacitracin and AgNCs, further proving they had a strong physical-chemical connection with each other, and the way that AgNCs could disperse uniformly in the bacitracin matrix material [31].

In the degeneration stage (Figure 3F, Table 1), the initial degradation temperature (T_onset_) of bacitracin (168.61 °C) was higher than that of Bacitracin-AgNCs (155.89 °C). This phenomenon might be due to the fact that the silver clusters began to degrade with increasing temperature, which made the integral Bacitracin-AgNCs degrade before the solitary bacitracin. According to the TG curves, the temperature values at the maximum decomposition rate (T_max_) of bacitracin and Bacitracin-AgNCs were 345.16 and 319.71 °C, respectively, demonstrating that the thermal stability of bacitracin was superior to that of Bacitracin-AgNCs at relatively low temperature; something that could be induced by the looser surface network structures of Bacitracin-AgNCs observed in FESEM images (Figure 3A,B). Meanwhile, the residual qualities of Bacitracin-AgNCs and bacitracin at 600 °C were 39.54 and 13.85%, respectively. The 25.69 wt% weight loss difference between the two revealed the presence of silver nanoclusters in Bacitracin-AgNCs [32]. Again, it also confirmed that the composite material Bacitracin-AgNCs had higher stability than that of bacitracin in general, because there were more residues of Bacitracin-AgNCs at 600 °C.

### 3.3. Antibacterial Activity of Bacitracin-AgNCs against S. flexneri

#### 3.3.1. Turbidity Test Results

**Figure 4:**
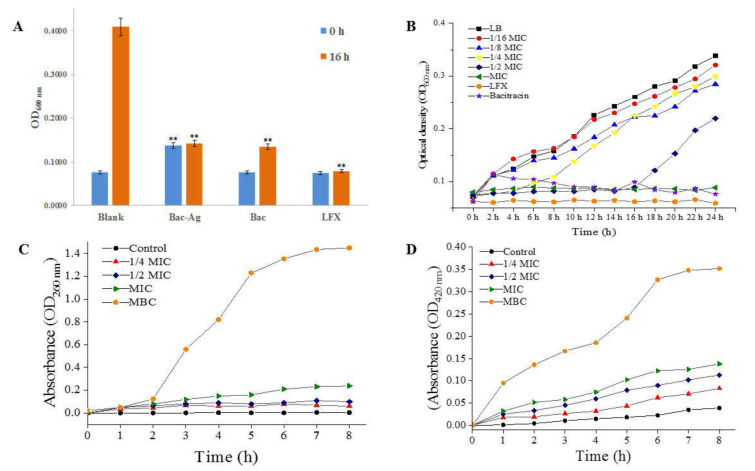
(**A**) Optical density at 600 nm (OD_600nm_) of bacteria in turbidity test. (**B**) The growth curves of *S. flexneri* treated with Bacitracin-AgNCs. (**C**) Effect of Bacitracin-AgNCs on nucleotide release and (**D**) cell membrane permeability of *S. flexneri*. ** Statistically significant differences (*p* < 0.05).

The result in Figure 4A shows that the OD_600_ value of control group without antibacterial agents increased significantly after culturing for 16 h, while the OD value of the bacitracin group increased slightly, indicating that bacitracin had a certain inhibitory effect on *S. flexneri*. By contrast, the OD values of the Bacitracin-AgNCs group and LFX group basically remained unchanged after 16 h culturing. The results reveal that Bacitracin-AgNCs had a similar antibacterial function to antibiotics LFX, and the antibacterial effect of Bacitracin-AgNCs was stronger than that of bacitracin alone.

#### 3.3.2. MIC, MBC of Bacitracin-AgNCs and Growth Curves

As shown in Table 2, the MIC and MBC of Bacitracin-AgNCs against *S. flexneri* were 0.031 and 4 mg/mL, respectively. The growth curves of *S. flexneri* treated with Bacitracin-AgNCs are shown in Figure 4B. Compared with the control, incubation with Bacitracin-AgNCs at concentrations below 1/8 MIC displayed no significant antibacterial effect on bacteria growth. Complete growth inhibition was obtained by Bacitracin-AgNCs at the MIC for 24 h culturing and the antibacterial effect of Bacitracin-AgNCs was higher than that of bacitracin at the same concentration.

### 3.4. Antibacterial Mechanism

#### 3.4.1. The Leakage of Intracellular Nucleotide

The membrane was destroyed and the intracellular components were released after bacterial membrane was exposed to antibacterial agents. Therefore, the membrane integrity could be well determined by measuring the OD values at 260 nm of supernatant. In Figure 4C, compared with the control, the absorbance of cell supernatant at 260 nm was slightly increased with Bacitracin-AgNCs at concentrations below 1/2 MIC, while the absorbance increased significantly with Bacitracin-AgNCs at the MIC and MBC. In the second hour, the values began to increase rapidly. After 6 h, the curves tended to be flat and the values reached a plateau. The results show that Bacitracin-AgNCs dealt significant damage to cell membrane and that, subsequently, intracellular components were released, with the damage being dose- and time-dependent.

#### 3.4.2. Effect of Bacitracin-AgNCs on *S. flexneri* Cell Membrane Permeability

After treatments of different concentrations of Bacitracin-AgNCs on *S. flexneri* for 8 h, the absorbance values of cells at 420 nm increased significantly (Figure 4D), while the *o*-nitrophenol production of the control group remained at a low level. From the result, Bacitracin-AgNCs increased the membrane permeability of *S. flexneri*, resulting in the leakage of key cellular enzymes.

#### 3.4.3. CLSM Observation of Bacitracin-AgNCs on *S. flexneri* Cell

**Figure 5:**
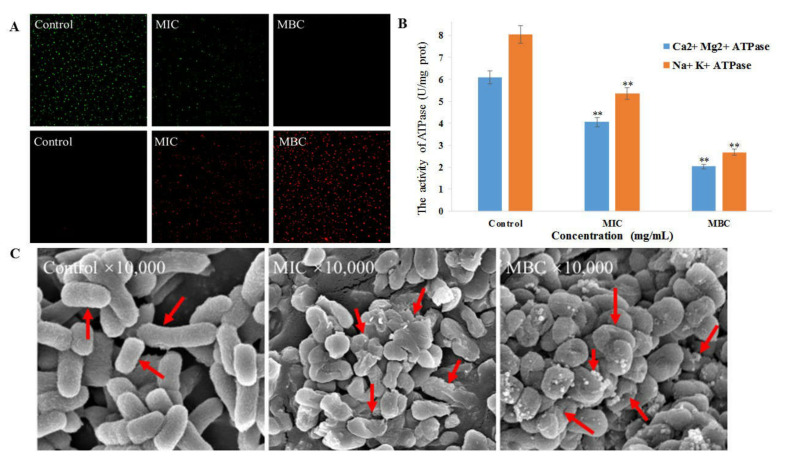
(**A**) CLSM images of *S. flexneri* cells stained with AO/EB. (**B**) The effect of Bacitracin-AgNCs on the activity of Ca^2+^ Mg^2+^- and Na^+^ K^+^-ATPase of *S. flexneri*. (**C**) FESEM images of *S. flexneri* following Bacitracin-AgNCs treatment. ** Statistically significant differences (*p* < 0.05).

The results of living/dead staining of *S. flexneri* cells are shown in Figure 5A. In the control group, *S. flexneri* cells exhibited uniform green fluorescence, almost no red fluorescence was observed, indicating that unprocessed cells confined in a complete normal condition. When the cells were exposed to Bacitracin-AgNCs at MIC, the green fluorescence decreased while the red fluorescence increased, demonstrating the membrane permeability of the treated group was reinforced compared with the control. A large amount of red fluorescence was found of *S. flexneri* cells treated with Bacitracin-AgNCs at MBC, and the green fluorescence became weak, showing the bacterial membrane was severely damaged.

#### 3.4.4. Cellular Na^+^ K^+^- and Ca^2+^ Mg^2+^-ATPase Activity

The effects of Bacitracin-AgNCs on two representative ATPase activities of *S. flexneri* cells are shown in Figure 5B. Compared with the control group, the intracellular ATPase activities decreased significantly with the increasing concentration of bacteriostatic agents, proving that Bacitracin-AgNCs could effectively inhibit the activity of endoenzyme which played an important role in the growth of *S. flexneri* cells.

#### 3.4.5. Field-Emission Scanning Electron Microscopy (FESEM) Analyses

FESEM was performed to further observe the surface morphology of *S. flexneri* cells of treated and control groups. In Figure 5C, the untreated cells present typical complete rod shapes and clear outlines with smooth surfaces. The surface of cells treated with Bacitracin-AgNCs at MIC exhibited collapse and shrinkage, and the cytomembrane was destroyed with the leakage of intracellular substances. After treatment with Bacitracin-AgNCs at MBC for 6 h, the cells were severely damaged and original rod-like morphology disappeared with serious atrophy, the Bacitracin-AgNCs were attached to the cells.

### 3.5. Effect of Bacitracin-AgNCs on Formation of S. flexneri Biofilm

**Figure 6:**
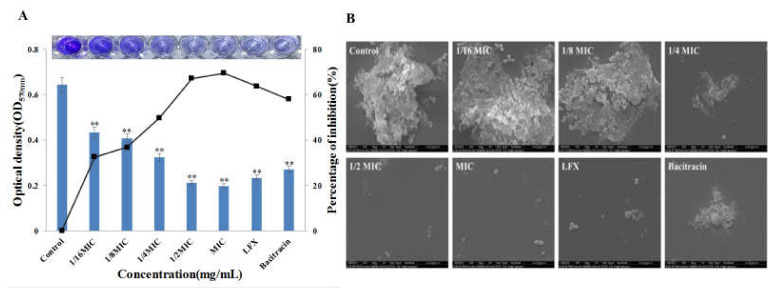
(**A**) Crystal violet quantitative assay for the effect of Bacitracin-AgNCs against *S. flexneri* biofilm formation. (**B**) ESEM images of the effects of Bacitracin-AgNCs on *S. flexneri* biofilm formation. ** Statistically significant differences (*p* < 0.05).

The inhibitory rates of Bacitracin-AgNCs on biofilm formation of *S. flexneri* and the ESEM observation results are shown in Figure 6. Inhibition of biofilm formation could be achieved by treatment with low concentrations (1/16 MIC, 1/8 MIC) of Bacitracin-AgNCs. The inhibition percentages were 49.72, 67.2 and 69.55% respectively after exposure to Bacitracin-AgNCs at 1/4MIC, 1/2MIC and MIC concentrations. The inhibition activity on biofilm formation enhanced with increasing concentration of Bacitracin-AgNCs.

In ESEM images, the biofilm structure was highly dense with many wrapped bacterial cells in the control group. By contrast, the biofilm became loose and scattered, and the wrapped cells decreased after addition of Bacitracin-AgNCs at 1/16 MIC and 1/8 MIC concentrations. The biofilm structure under treatment at 1/4 MIC and 1/2 MIC concentrations was damaged with the disappearance of extracellular polymers. The cell integrity of some bacteria was destroyed, and almost no visible biofilm structure was observed after Bacitracin-AgNCs treatment at the MIC level. Meanwhile, LFX and bacitracin also had a certain inhibitory effect on the formation of biofilm, but the action was inferior to that of Bacitracin-AgNCs at MIC. The results indicate that Bacitracin-AgNCs damaged the biofilm integrity.

## 4. Discussion

Bacitracin is a polypeptide antibiotic that has bacteriostasis to not only Gram-positive bacteria, but also negative cocci, diplococcus pneumoniae, staphylococcus and spirochete. As a biological template, it can easily combine with metal ions to produce nanomaterials with stronger antibacterial effect. In this study, the UV-VIS absorption peak of the synthesized Bacitracin-AgNCs at 414 nm indicates that the required nanomaterial was successfully prepared. The optimum excitation and emission wavelengths were 384 nm and 460 nm respectively, which are consistent with the reported study [15] showing similar fluorescence properties. The results of FT-IR and XPS analyses depicted the functional groups and energy spectrum on the composite in detail, and the hydrophilicity of Bacitracin-AgNCs could be explained by hydroxyl groups on its surface [33].

Peptides and antibiotics containing peptides can inhibit microbial growth via inhibition of the synthesis of cell wall, DNA and protein, and disrupting the integrity of the microbial membrane [34]. Previous research [35] has revealed that bacitracin can inhibit the biosynthesis of *Staphylococcus aureus* cell wall by combining with divalent metal ions such as Cu, Ni, Co, Zn and Mn, thereby inhibiting the growth of *Staphylococcus aureus*. Additionally, the antibacterial effect of the synthetic complex was better than that of pure bacitracin. The novel silver nanoparticles synthesized by a green method have broad-spectrum antibacterial properties against foodborne pathogens, involving *Escherichia coli*, *Pseudomonas aeruginosa*, *Listeria monocytogenes*, *Staphylococcus aureus*, *Vibrio parahaemolyticus* and *Salmonella typhimurium* [36]. In this study, the composite formed by the binding of bacitracin and silver ions also showed significant inhibitory action against *S. flexneri*. These results indicate that bacitracin can bind to metal ions well, and the synthesized Bacitracin-AgNCs is a new antibacterial agent with efficient antibacterial properties. Growth curve and turbidity assays analyses further showed that the enhancement of the antibacterial effect of Bacitracin-AgNCs was related to the optical density decrease of *S. flexneri*. Wang et al. [15] revealed a similar effect of bacitracin silver ion complexes on the growth of *Staphylococcus aureus*, where higher concentrations of antibacterial agents led to lower growth rates.

The cell membrane is an important active structure with barrier function, which plays a vital role in maintaining metabolism and energy conversion, and it is between cytoplasm and extracellular medium [37]. Many antibacterial drugs inhibit bacterial growth by a membrane injury mechanism, including changing cell membrane permeability, destroying cell membrane integrity and inducing leakage of cell contents [38,39]. When bacteria are exposed to biocides, the membrane may be impaired, thus resulting in the release of intracellular DNA, RNA, hot materials, protein, and some salt ions such as K^+^ and phosphate from the cells. The released nucleotides have a strong UV absorption ability at 260 nm, so that, consequently, the integrity of the cell membrane can be evaluated in accordance with the UV absorption values of bacterial suspension at 260 nm [40]. In Figure 4C, the absorbance values increase rapidly at the second hour and remain stable after six hours. This phenomenon might be due to the breakdown of a bacterial lipopolysaccharides layer induced by antibacterial agents, leading to the rapid loss of cell ingredient in a short time [41]. In addition, *β*-galactosidase cannot pass through the complete bacterial membrane, but can be detected by leaking to the outside of the bacteria when the cell membrane is damaged [42]. Our results show that the addition of Bacitracin-AgNCs caused harm to the bacterial membrane, the loss of its barrier function and obstacles to bacterial growth.

AO and EB are frequently used for the cell living/dead staining, and they were used to further estimate the effect of Bacitracin-AgNCs on bacterial membrane permeability in this study. AO can penetrate cell membrane and embed in the nuclear DNA to emit green fluorescence, while EB can only pass through the damaged cell membrane and stain dead cells when accompanied by red fluorescence [43]. In our study, the control group showed complete green fluorescence, whereas the agents-treated groups showed a certain degree of red fluorescence, indicating that Bacitracin-AgNCs increased the permeability of *S. flexneri* cell membrane in a dose-dependent manner (Figure 5A). Zawadzka et al. [44] also reported that the composite material of TiO_2_ coating and silver nanoparticles changed the permeability of *Staphylococcus aureus* membrane, and caused damage to DNA by penetrating into the cells of bacteria.

Bacitracin-AgNCs can act on *S. flexneri* cells to destroy the membrane and lead to cell death, so does it affect the intracellular ATPase in the cell? In our study, Bacitracin-AgNCs significantly inhibited the activity of Na^+^ K^+^- and Ca^2+^ Mg^2+^-ATPase in *S. flexneri*, which may be due to the rapid termination of intracellular biosynthesis resulting from cell membrane depolarization [45]. Intracellular ATPase is essential for cell growth, among them, Na^+^ K^+^-ATPase is an immanent sodium pump in many eukaryotic cell membranes, which plays an important role in establishing and maintaining intracellular high K^+^ and low Na^+^ concentrations. Moreover, the establishment of Na^+^ electrochemical gradient on the plasma membrane is also crucial for some cell functions, such as pH regulation and nutrient absorption [46]. A similar study has reported the inhibitory effects of *ε*-polylysine and nisin on Na^+^ K^+^- and Ca^2+^ Mg^2+^-ATPase activity of *Bacillus subtilis* [45]. Furthermore, FESEM images showed that the cell morphology of *S. flexneri* changed significantly after being treated with Bacitracin-AgNCs, and some small particles were attached to the cells, which may be the synthetic nanomaterials. The result further proves the damage to the cell membrane structure and the leakage of intracellular substances. Juan et al. [36] observed that small particles which might be AgNPs adhered to the surface of *Vibrio parahaemolyticus* cells, and that this phenomenon could result in changes in the cell’s morphology and membrane properties. Due to the adhesion of AgNPs to bacterial cells, a series of pores and holes were formed on the cells surface, causing the dissolution and death of *Staphylococcus aureus* [44]. In general, Bacitracin-AgNCs caused irreversible damage to cell morphology and membrane permeability, so that it seriously inhibited the growth of *S. flexneri*.

Biofilm is a widespread multicellular form in the process of bacterial growth, which can provide protection and the potential for homeostasis [47]. Relevant statistic shows that 80% of bacterial infections in humans are related to biofilm. That is, biofilms have very important barrier effects on cells. Therefore, in this research, we also explored whether Bacitracin-AgNCs can affect the formation of biofilm at sub-MICs concentrations. In other words, we verified that Bacitracin-AgNCs at sub-MICs had a negative effect on the growth of *S. flexneri* and a dose-dependent inhibitory effect on biofilm formation through a crystal violet quantitative experiment. ESEM observations further confirmed that Bacitracin-AgNCs destroyed the whole biofilm structure and greatly reduced the living cell counts in biofilms. These results are consistent with the description of a previous study [48], where it was observed that the presence of AgNCs and the increase of its concentrations impeded biofilm formation of *Escherichia coli* and *Staphylococcus aureus*. Bacitracin-AgNCs hindered the normal growth and reproduction of bacteria by breaking the integrity of the biofilm. 

## 5. Conclusions

In summary, a new type of Bacitracin-AgNCs nanomaterial was synthesized in a novel and eco-friendly manner. We used a variety of instruments to characterize the synthesized nanoclusters, and further studied the antibacterial activities and mechanisms of Bacitracin-AgNCs against *S. flexneri*, a food-borne pathogen. It can be seen that Bacitracin-AgNCs unfolded potent antibacterial activity against *S. flexneri* and could inhibit the growth of bacteria at low concentrations. Bacitracin-AgNCs could destroy the integrity of membrane, resulting in visible morphological changes and the leakage of intracellular nucleotides, before cell proliferation was finally blocked. This study contributes to the interpretation of the antimicrobial mechanisms of Bacitracin-AgNCs as a new antibacterial agent against *S. flexneri* and lays the foundation for further research of its application in the food industry.

## Figures and Tables

**Table 1:** Thermal properties results of bacitracin and Bacitracin-AgNCs.

Code	T_onset_ (°C)	T_max_ (°C)
Bacitracin	168.61	345.16
Bacitracin-AgNCs	155.89	319.71

**Table 2:** Minimum inhibitory concentration (MIC) and minimum bactericidal concentration (MBC) of Bacitracin-AgNCs against *S. flexneri*.

Bacteria	Concentrations of Bacitracin-AgNCs (mg/mL)										
	0	0.015625	0.03125	0.0625	0.125	0.25	0.5	1	2	4	8
*Shigella flexneri*	++	++	+	+	+	+	+	+	+	-	-

Note: “++”: means obvious bacterial growth, “+” means no visible bacterial growth, “-” means no visible bacterial colonies on the surface of SS agar plates.

## Data Availability

All data used and analyzed in this study are shown within the study. If further data are required, it can be available by the corresponding author under reasonable request.
